# Bevacizumab for radiation necrosis following radiotherapy of brain metastatic disease: a systematic review & meta-analysis

**DOI:** 10.1186/s12885-021-07889-3

**Published:** 2021-02-16

**Authors:** Muhammad Khan, Zhihong Zhao, Sumbal Arooj, Guixiang Liao

**Affiliations:** 1 Department of Oncology, Shenzhen People’s Hospital, The First Affiliated Hospital of Southern University of Science and Technology, Shenzhen, 518020 People’s Republic of China; 2 Department of Oncology, First Affiliated Hospital of Anhui Medical University, Hefei, Anhui Province People’s Republic of China; 3 Department of Nephrology, Shenzhen People’s Hospital, Second Clinical Medicine Centre, Jinan University, Shenzhen, People’s Republic of China; 4 Department of Biochemistry, University of Sialkot, Sialkot, Pakistan

**Keywords:** Bevacizumab (BV), Radiation necrosis (RN), Dexamethasone (Dex), MRI imaging, Adverse events

## Abstract

**Background:**

Radiotherapy is the mainstay of brain metastasis (BM) management. Radiation necrosis (RN) is a serious complication of radiotherapy. Bevacizumab (BV), an anti-vascular endothelial growth factor monoclonal antibody, has been increasingly used for RN treatment. We systematically reviewed the medical literature for studies reporting the efficacy and safety of bevacizumab for treatment of RN in BM patients.

**Materials and methods:**

PubMed, Medline, EMBASE, and Cochrane library were searched with various search keywords such as “bevacizumab” OR “anti-VEGF monoclonal antibody” AND “radiation necrosis” OR “radiation-induced brain necrosis” OR “RN” OR “RBN” AND “Brain metastases” OR “BM” until 1st Aug 2020. Studies reporting the efficacy and safety of BV treatment for BM patients with RN were retrieved. Study selection and data extraction were carried out by independent investigators. Open Meta Analyst software was used as a random effects model for meta-analysis to obtain mean reduction rates.

**Results:**

Two prospective, seven retrospective, and three case report studies involving 89 patients with RN treated with BV were included in this systematic review and meta-analysis. In total, 83 (93%) patients had a recorded radiographic response to BV therapy, and six (6.7%) had experienced progressive disease. Seven studies (*n* = 73) reported mean volume reductions on gadolinium-enhanced T1 (mean: 47.03%, +/− 24.4) and T2-weighted fluid-attenuated inversion recovery (FLAIR) MRI images (mean: 61.9%, +/− 23.3). Pooling together the T1 and T2 MRI reduction rates by random effects model revealed a mean of 48.58 (95% CI: 38.32–58.85) for T1 reduction rate and 62.017 (95% CI: 52.235–71.799) for T2W imaging studies. Eighty-five patients presented with neurological symptoms. After BV treatment, nine (10%) had stable symptoms, 39 (48%) had improved, and 34 (40%) patients had complete resolution of their symptoms. Individual patient data was available for 54 patients. Dexamethasone discontinuation or reduction in dosage was observed in 30 (97%) of 31 patients who had recorded dosage before and after BV treatment. Side effects were mild.

**Conclusions:**

Bevacizumab presents a promising treatment strategy for patients with RN and brain metastatic disease. Radiographic response and clinical improvement was observed without any serious adverse events. Further class I evidence would be required to establish a bevacizumab recommendation in this group of patients.

## Introduction

Brain metastasis (BM) is the most common adult intracranial disease, and it is diagnosed in approximately 20 to 30% of cancer patients [1–3]. The most common primary tumor metastasizing to the brain is lung cancer (up to 50%), followed by breast cancer (up to 25%), melanoma (up to 20%), and to a lesser extent, renal cell carcinoma, colorectal cancer, and others [1–4]. Nonetheless, the incidence and frequency of BM is growing as newer systemic and immunotherapeutic agents are entering the treatment paradigm of these primary cancers [5–9]. Patients are living longer and are more prone to experience BM in their lifetime.

Depending on various prognostic factors, management of BM may involve surgical resection and/or radiation therapy in the form of stereotactic radiosurgery (SRS), whole brain radiotherapy, or a combination of two [1, 10–13]. A surge has been witnessed in the use of radiosurgery in BM patients with the approval of various targeted and immunotherapeutic agents for the management of primary sites of systemic cancers [6–9, 14]. Targeted agents after SRS for the brain have also been continued and have prolonged survival outcomes for patients with BM [6, 7, 9, 15, 16]. Radiation therapy has long been associated with the development of radiation necrosis (RN) in patients with intracranial disease [17–21]. The rate of RN following radiotherapy or radiosurgery has been estimated at 10–15% [17–21]. RN is considered as a dose-limiting toxicity for SRS [20, 21]. An increased incidence of RN has also been reported with a combination of SRS and systemic agents [22, 23]. In fact, the benefits of synergism from a combination of radiation and targeted agents are weighed against RN toxicity [7, 22, 23]. Hence, the management of RN takes a center stage in patients with intracranial disease.

Corticosteroids have long been the mainstay of RN treatment. It inhibits the pro-inflammatory response that promotes radiation necrosis and provides symptomatic relief via edema reduction, but long-term use is associated with serious side effects [19]. Surgery has also been used for resectable progressive RN, which can relieve mass effects and it also provides an opportunity to study tissue samples for diagnosis. However, persistent edema may need close monitoring for weeks [19, 24]. Another treatment strategy employed is hyperbaric oxygen therapy (HBOT) [25]. It can increase oxygen concentration to stimulate angiogenesis, restore blood supply to necrotic lesions, and accelerate healing. It has also shown improvement in RN symptoms alone or in combination with Endostar (a recombinant endostatin product) [25]. Laser interstitial thermal therapy (LITT) has been demonstrated to relieve RN symptoms, reduce progression, and improve survival in patients with RN and brain metastases [26, 27]. It has also been used to complement RN surgery [24]. Bevacizumab (BV) has also made it a treatment paradigm for RN [28–30]. Recent clinical trials have shown encouraging results [31–33].

Bevacizumab, an anti-VEGF monoclonal antibody, has been evaluated for RN treatment [28–30]. Its use in RN stems from the fact that RN tissues have elevated levels of VEGF [34, 35]. Radiotherapy induces vasogenic edema and ischemia, resulting in hypoxia that leads to the induction of hypoxia-inducible factor 1α (HIF1α) [34–38]. HIF1α upregulates VEGF through astrocytes and endothelial cells [36, 38]. White matter around necrotic areas has been identified as the main VEGF up-regulating site [36]. Immunohistochemistry (IHC) of RN surgical samples has confirmed increased levels of VEGF in reactive astrocytes surrounding the core of necrotic tissue [37]. VEGF is an important regulator of angiogenesis, leading to increased vascular permeability, damage to the blood-brain barrier (BBB), and ensuing brain edema [39]. Bevacizumab reduces vascular permeability and alleviates blood-brain barrier damage and brain edema through its binding to VEGF [28, 35, 39].

Several studies have reported the efficacy of BV in the treatment of RN diagnosed in primary brain tumor, metastatic, and patients with nasopharyngeal carcinoma (NPC) [31–33, 40–58]. Two randomized controlled trials have shown its efficacy over placebo or corticosteroid-receiving patients, without any increase in toxicity in primary brain tumors and NPC patients [31, 32]. Recently, a prospective phase II clinical trial has revealed efficacy of BV in patients with metastatic brain disease who have RN [33]. However, the majority of studies had included patients without differentiating for their intracranial disease type [50–54]. Here, we conducted a systematic review to gather evidence of the clinical efficacy of BV for patients with metastatic brain disease who have RN.

## Methods & materials

Preferred Reporting Items for Systematic Reviews and Meta-Analyses (PRISMA) guidelines were rigorously followed [59].

### Inclusion criteria

#### Patients & study types

Studies reporting the efficacy of bevacizumab for radiation necrosis occurring in patients with brain metastases after undergoing radiotherapy for intracranial disease.

#### Types of interventions

Bevacizumab

#### Outcomes of interest

Outcomes of prime interest were: radiographic response; edematous volume reductions on magnetic resonance imaging (MRI); and clinical improvement such as improvement/resolution of neurological symptoms and signs, increase in Karnofsky Performance Status (KPS) score, and decrease in dosage or discontinuation of dexamethasone. The secondary outcomes of interest were recurrence and safety outcomes, including adverse events.

### Search strategy

#### Databases

PubMed, Medline, EMBASE, and the Cochrane library were searched until 1st Aug 2020. Various search terms such as “bevacizumab” OR “Anti-VEGF monoclonal antibody” AND “Radiation necrosis” OR “Radiation induced brain necrosis” OR “RN” OR “RBN” AND “Brain metastases” OR “BM” etc., were employed. Language was restricted to English. Furthermore, references of the retrieved studies were also inspected for more relevant literature.

#### Study selection

Relevant studies obtained from databases were imported into Endnote X9.3 software for organization and screening. Duplicates were removed and titles and abstracts were thoroughly screened. Studies were selected according to the aforementioned inclusion criteria. In situations of discrepancies, other authors were consulted.

### Data extraction

“The Cochrane Collaboration Data Collection form-RCTs and non-RCTs” was modified according to our requirements and used for recording data. The extracted data included general characteristics/attributes of the studies and participants and the main outcomes of interest. The characteristics of the studies recorded were the first author, publication year, period of recruitment, research design, institute of research, number of participants, and follow-up time. The recorded attributes of participants included age, sex, presenting symptoms, KPS, dexamethasone use, and adverse events.

Furthermore, outcomes of interest, including radiographic response, RN volume reduction on MRI images, clinical improvement, and safety. Scrutiny and examination of eligible studies was accomplished with full text reading by two independent reviewers (M.K. and Z.Z).

### Assessment of risk for bias

Quality assessment was carried out using the Reporting Checklist for Authors developed by The Meta-analysis Of Observational Studies in Epidemiology (MOOSE) Group [60].

### Statistical analysis

Descriptive statistics, including frequency, percentage, mean, median, range, and standard deviation, were calculated with Microsoft Excel for Mac 2019 v16.43. Mean reduction rates were directly extracted from the studies or indirectly via Engauge Digitizer. The weighted mean and standard deviation was estimated according to the methods described in the Cochrane Handbook for Systematic Reviews of Interventions version 6.0 [61, 62]. Pooled estimates (weighted mean and confidence interval) was obtained with Open Meta Analyst software, which uses the R package “metafor” for meta-analysis [63–65]. The pooled mean was estimated using a continuous random effects model with the DerSimonian-Laird method [66]. Heterogeneity was assessed using the *I*^*2*^ test. *I*^*2*^ values of 25, 50, and > 50% were considered as low, moderate, and high heterogeneity [67]. *P* < 0.05 was considered statistically significant.

## Results

**Fig. 1:**
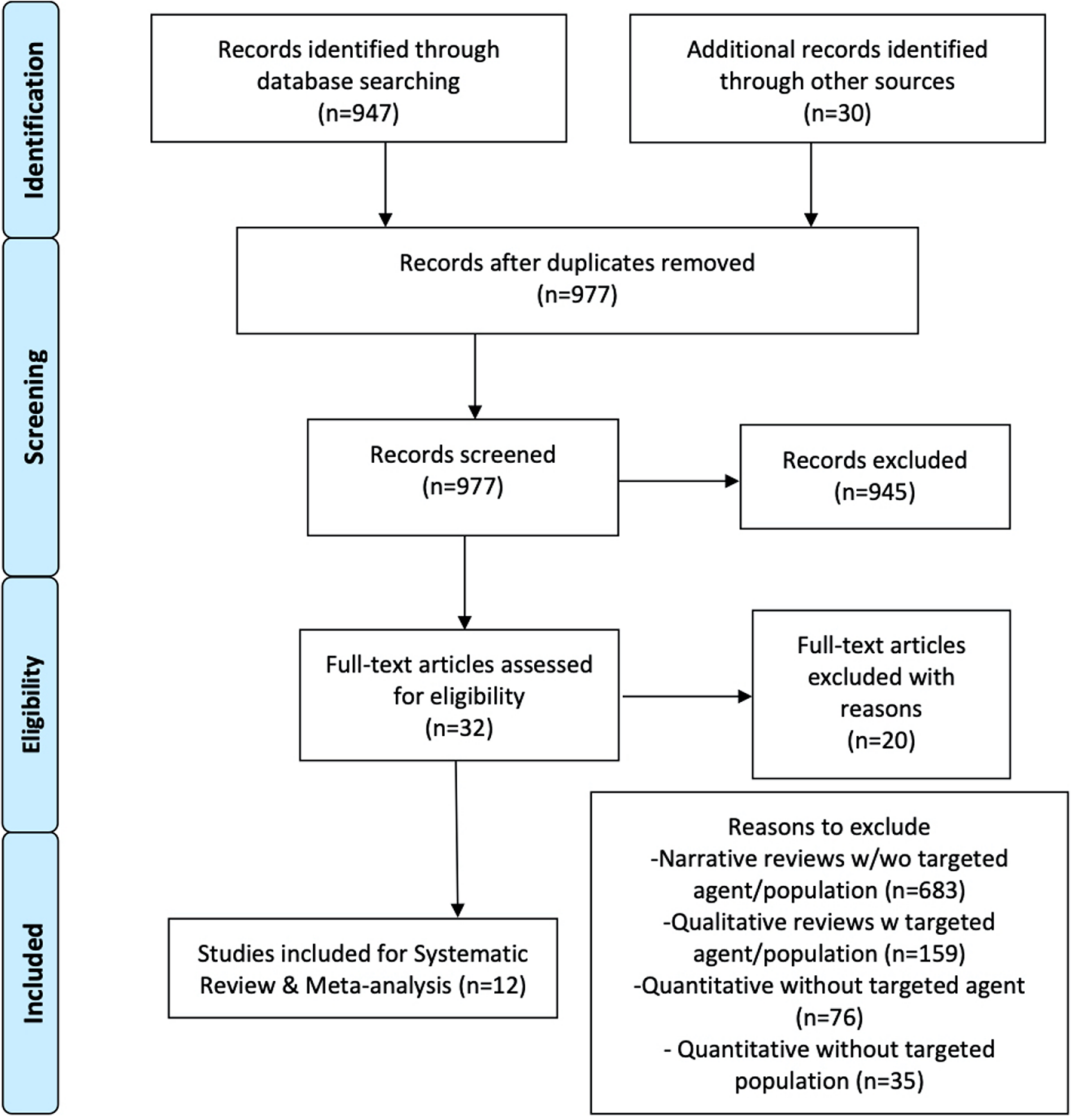
PRISMA flow diagram of research strategy and study selection

Overall, two prospective studies, seven retrospective studies, and three case reports involving 89 patients with RN treated with BV were obtained following the research strategy and study selection process [33, 48–58]. The PRISMA flow diagram for the same is illustrated in Fig. 1. Among them, 39 patients were male and 50 were female (Table 1). Lung (54, 61%) and breast (12, 14%) cancers constituted the main primary pathology for BM. All patients had developed RN after receiving radiation therapy to the brain [33, 48–58]. Stereotactic radiotherapy (SRT) (37, 33%), which is SRS delivered in fractions, was the main component of treatment delivered after BM development, followed by single-dose SRS (26, 23%) and whole-brain radiotherapy (WBRT) (22, 19%). SRS was also the main radiation strategy given as radiation boost after conventional radiotherapy (24, 21%) [33, 48–58]. The time duration from radiotherapy induction to RN development was reported in most studies as the time from RT to RN diagnosis and, in a few studies, as RT to BV induction (Table 1). The mean time from RT to RN diagnosis ranged between 6.5 and 19 months, and for RT to BV induction was between 4.6 and 11 months [33, 48–58]. All the studies had used various combinations of diagnostic procedures to determine the RN diagnosis, including MRI, magnetic resonance spectroscopy (MRS), methionine positron emission topography (MET-PET scans), and biopsy/pathology [33, 48–58]. Differentiation between disease progression and RN diagnosis was based on the imaging guidelines reported in previous studies [68–74]. The imaging characteristics are outlined in Table 2. Most common dose of BV used were 5–10 mg/kg [49–54, 57]. Other doses applied ranged from as low as 1 mg/kg to as high as 15 mg/kg [33, 48–58]. The timing of BV induction ranged from every 2 weeks to every 6 weeks [33, 48–58]. The mean number of treatment cycles completed by patients ranged from two to six cycles. Follow up time also varied from 3.3 to 22.7 months. The details are illustrated in Table 1.

**Table 1:** General characteristics of the included studies

Study	Study design & period	Location	No	Age	M	F	Primary pathology	Radiation	RN Diagnosis	RT to RN Diagnosis	RT to BV Tx	BV dosage	No. of cycles	Follow Up
Wang, et al. (2012) [50]	Retrospective Mar 2010 - Jan 2012	Huashan Hospital, Fudan University, Shanghai, China	5	65	4	1	Colon 3 Lung 2	EBRT/SRS/FSRT	MRI, MRS, PET		4.6	7.5 mg/kg q2 week	2–6	6
Boothe, et al. (2013) [51]	Retrospective 3-year	Memorial Sloan- Kettering Cancer Center, New York, USA	11	58	4	7	Breast 5 NSCLC 6	WBRT/SRS	MRI, biopsy, PET	12.4	59.6 days	7.5 mg/kg q3w (1) 10 mg/kg q2w (8) 15 mg/kg q4/6w (2)	6	3.3
Furuse, et al. (2013) [52]	Retrospective Jan 2009 - Oct 2010	Osaka Medical College, Takatsuki, Osaka, Japan	3	62	1	2	Unknown	SRS	MRI, MET-PET		11 (median)	5 mg/kg q2w	3	14.4
Yonezawa, et al. (2014) [53]	Prospective Nonrandomized Jun 2010 - Sep 2011	Kizawa Memorial Hospital, Minokamo, Japan	2	52.5	1	1	Lung	WBRT/SRS/SRT	MRI, MET-PET	19		5 mg/kg q2w	6	
Sadraei, et al. (2015) [54]	Retrospective Jul 2007 - Jun 2012	Cleveland Clinic, Cleveland, Ohio, USA	17	55.7	5	12	lung (9), breast (4), rectal (1), melanoma (1), NSTC (1), FT (1)),	WBRT/SRS	MRI, PET, biopsy	16.9 10.1		5/7.5/10/15 mg/kg q2/3w	6	8
Zhuang, et al. (2015) [55]	Retrospective Jun 2011 - Dec 2014	Tianjin Cancer Hospital, Tianjin, China	14	56	6	8	Lung 11, Breast 1, Lymphoma 1, Gastric cancer 1	RT	MRI, PET, pathology			5 mg/kg q3-4w	3	12
Xiang-Pan, L., et al. (2015) [49]	Retrospective	Wuhan, China	1	60		1	Lung	WBRT/SRS	MRI	12		7.5 mg/kg q3w	2	
Alessandretti, et al. (2013) [48]	Retrospective	Hospital São José, São Paulo, Brazil	2	49.5		2	Melanoma	WBRT/SRS	MRI	11.5		5,7.5 mg/kg q6/4w		
Zhuang, et al. (2019) [33]	Prospective II CT Dec 2016 - Feb 2019	Tianjin Cancer Hospital, Tianjin, China	21	55 (median, range 43–70)	11	10	Lung 17 Breast 2 Kidney cancer 2	SRT	MRI	17.6		1 mg/kg q3w	3	22.7
Tanigawa, et al. (2019) [56]	Retrospective	Kagoshima University, Kagoshima, Japan	4	61.25	2	2	Lung	STI (stereotactic irradiation)	MRI	7.75		15 mg/kg q3–4w		
Ma, et al. (2017) [57]	Retrospective	Zhejiang University, Hangzhou, China	2	62		2	NSCLC	SRS	MRI	6.5		5 mg/kg q2w/7.5 mg/kg q3w	2.5	9
Glitza, I. et al. (2017) [58]	Retrospective	The University of Texas MD Anderson Cancer and Baylor College of Medicine, Houston, Texas, USA	7	57	5	2	Melanoma	SRS/WBRT	Surgery, MRI, pathology	8.14		5, 7.5, 10 mg/kg	3.7	
This study	Systematic review		89		39	50						1-15 mg/kg q2-6w	2–6	

*Abbreviations*: *CT* Clinical trial, *No* No of patients, *M* Male, *F* Female, *WBRT* Whole brain radiotherapy, *SRS* Stereotactic radiosurgery, *SRT* Stereotactic radiotherapy, *EBRT* External beam radiotherapy, *RT* Radiotherapy, *FSRT* Fractionated stereotactic radiotherapy, *RN* Radiation necrosis, *BV* Bevacizumab, *Tx* Treatment, *NSCLC* Non-small cell lung cancer, *FT* Fallopian tube, *NSTC* Non-seminomatous testicular cancer, *MRI* Magnetic resonance imaging, *PET* Positron, emission topography, *q2w* Every 2 weeks

**Table 2:** Imaging characteristics for diagnosis of radiation necrosis

Imaging Technique	Characteristics
MRI	-Contrast enhancement pattern (soap bubble or Swiss cheese pattern, etc.), -Location of enhancement (periventricular, corpus callosum, midline crossing, subependymal spread), -Multiplicity (single/multiple), -Distance from primary tumor site (ipsilateral/contralateral)
MRS	-Decreased peaks in Cho, NAA and Cr, -Low Cho/Cr values -Elevated Lip-Lac/Cho
PET	-No uptake of radionuclides

*Abbreviations*: *MRI* Magnetic resonance imaging, *MRS* Magnetic resonance spectroscopy, *PET* Positron emission topography

### Measurement of MRI changes and calculation of reduction rate

Slight variations were noticed in methods for assessing the volume calculation and reduction rate on MRI images among the studies. Two studies estimated the area of lesion at the level of maximum bi-dimensional measurement according to McDonald’s criteria, and the difference was expressed as percent change from the baseline MRI profiles [50, 54, 75]. In some studies, the hyperintense area was manually outlined, measured, and summed across slices and was multiplied by the layer thickness to calculate the total lesion area, but the reduction rate was estimated differently [33, 51–53, 55]. Volume reduction was obtained by subtracting of post-treatment from pre-treatment volume, dividing post-treatment by pre-treatment volume, and the following formula: volume before BV – volume after BV / volume before BV [33, 51–53, 55]. Zhuang et al. calculated the edema index as: EI = volume of (edema + necrosis)/volume of necrosis [33, 55]. For T1 MRI, changes in the signals were measured in three different areas in the strengthening region of necrosis and compared to the white matter signal value of the same MRI to obtain a ratio that was used to express the reduction rate as the difference between pre- and post-treatment [33, 55]. We calculated the difference from the graphs available in their studies.

### Patients characteristics

Ten studies reported individual patient characteristics and treatment-related data for 54 patients with RN [48–54, 56–58]. The details are outlined in Table 3. These patients consisted of 22 male and 32 female patients, and their average age was 58 years. The mean time from RT to RN diagnosis was 11.7 months and from RT to induction of BV treatment was 15.5 months [48–50, 52–54, 56–58]. BV dosage ranged from 5 mg/kg to as high as 15 mg/kg, every 2 weeks to every 6 weeks for an average of 5.7 treatments [48–50, 52–54, 56–58]. Three studies also provided treatment durations for each patient [48, 51, 57]. The mean BV treatment duration averaged at 3.29 months [48, 51, 57]. Neurological symptoms, such as headache, visual disturbances, seizures, limb weakness, etc., have been reported in nine studies [33, 48, 51–58]. Five studies reported adverse events after BV for individual patients [33, 54–56, 58]. Detailed information is provided in Table 3.

**Table 3:** Individual characteristics and treatment outcomes for RN patients

Study	Age	Sex	Primary histology	Radiation	Dose	RN site	Last RT to RN diagnosis	RT to BV Tx	BV dosage (mg/kg)	No of treatments	Treatment Duration (months)	Volume reduction on T1W-Gd-enhanced MRI	Volume reduction on T2W FLAIR MRI	Pre-Tx KPS	Post-Tx KPS	KPS increase	Pre-Tx Dex (mg)	Post-Tx Dex (mg)	Dex reduction (mg)	Presenting Symptoms	Improvement	Adverse events
Wang, et al. (2012) [50]	70	M	colon	SRS	17 Gy	L temporal		6				0	0	30	30	0	15	12.5	2.5		Improved	
	71	M	colon	EBRT	36 Gy	L frontal		4				65	10	40	80	40	15	5	10		Improved	
	71	M	Lung	FSRT	31.5 Gy/3f	L occipital		7				87	78	50	90	40	15	0	15		Improved	
	67	F	colon	EBRT SRS	39 Gy/10 f 16 Gy	R frontal		1				52	78	50	90	40	15	0	15		Worsened	
	46	M	Lung	EBRT SRS	30 Gy/13 f 16 Gy	L occipital		5				50	30	60	80	20	12.5	5	7.5		Improved	
Boothe, et al. (2013) [51]	58	M	NSCLC	WBRT SRS	37.5 25/25	R frontal/L temporal			10q2w		2.3	38/64	3/36				8	0	8	None	Improved	
	50	F	Breast	SRS	30	R occipital			10q2w		2.3	82	75				2	0.3	1.7	Visual field disturbance, headaches	Stable	
	27	F	Breast	WBRT SRS	37.5 18/21/21	L frontal/L temporal/R parietal			10q2w		1.4	38/64/91	70/67/63.5				4	0	4	Seizures	Improved	
	79	F	NSCLC	SRS	18	R parietal			10q2w		0.5	82	60				0.2	0	0.2	Lower left leg weakness	Improved	
	67	F	Breast	WBRT SRS	30 18	Cerebellum			10q2w		14.3	73	72							Headaches, lower leg weakness	Stable	
	54	F	Breast	WBRT SRS	37.5 15	R frontal			10q2w		3.9	21	44.6							Left arm weakness	Improved	
	67	M	NSCLC	SRS	30	R frontal			15q6w		2.8	10	3				24	0	24	Seizures, left sided weakness	Improved	
	50	F	Breast	WBRT SRS	35 21	R frontal			7.5q3w		1.4	91	46				20	0	20	Fatigue, lethargy, facial asymmetry	Improved	
	67	F	NSCLC	SRS	21	L occipital			102w 4 w			89	84.5				6	0	6	Confusions, visual hallucinations	Stable	
	73	M	NSCLC	SRS	21	L parietal			102w			96	54.6				8	2	6	Seizure, right sided hemiparesis	Improved	
	63	M	NSCLC	SRS	21	L occipital			15q4w		1.8	100	77				8	0	8	Imbalance, right sided tinnitis	Resolved	
Furuse, et al. (2013) [52]	57	F	unknown	SRS		frontal		5					73.4	40	60	20					Improved	
	74	F	unknown	SRS		frontal		47					74.4	60	60	0					Resolved	
	55	M	unknown	SRS		frontal		49					77.5	80	90	10					Stable	
Yonezawa, et al. (2014) [53]	54	M	Lung	WBRT SRS	30 20		15					55.9	88.9	60	70	10				Seizure, motor weakness	Improved	
	51	F	Lung	SRT	30/5 f		23					43.2	65	90	100	10				Headache, numbness	Improved	
Sadraei, et al. (2015) [54]	61	M	NSCLC	SRS	18 Gy	R posterofrontal cingulate	8		5q2w	8		35.2	92				24	0.5	23.5	Left sided weakness, gait problems	Improved	
	46	F	NSCLC	RT SRS	36.5 24	R cerebellar	17 11		5q2w	9		56.1	83.6							Y	Improved	
	62	M	NSCLC	SRS	18, 24, 24	Frontal, L temporal	16 4		7.5q3w	3		+ 37.8	+ 74.1				16	4	12	Y	Improved	
	59	F	NSCLC	WBRT SRS	44 24	R cerebellar	6 5					30.8	58.9							Y	Resolved	Proteinuria (bevacizumab held) grade 1
	58	F	NSCLC	WBRT SRS	40 24	R temporal L frontoparietal	58 53		10q2w	9		28.8	34.1				16	0	16	Y	Resolved	UTI (requiring holding of 1 treatment) grade 2
	46	F	NSCLC	WBRT SRS	37.5 18	L occipital	17 10		7.5q3w	10		18.5	48.2				8	0	8	Y	Improved	
	58	M	NSCLC	WBRT SRS	37.5 24	L parietal	11 9		15q3w	4		100	38.1				8	0	8	Y	Improved	
	63	F	NSCLC	SRS	18	Bithalamic L midbrain	18		7.5q3w	4		76.9	52.9				8	0	8	Y	No	
	55	F	Breast	WBRT SRS	37.5 24	L posterofrontal	14 6		10q2w	5		35.4	43.2				24	2	22	Y	Improved	
	58	F	Breast	WBRT SRS	37.5 18	R frontal	27 11		5q2w	3		64.7	26.7				8	8	0	Y	Improved	
	52	F	Breast	WBRT SRS	37.5 18	L cerebellar	7 5		10q2w	13		66.7	32.8				2	1	1	Y	Improved	
	58	F	Melanoma	WBRT SRS	37.5 24	L frontal	3 7		7.5q3w	2		82.4	74.9				6	4	2	Y	Resolved	DVT and PE grade 3
	39	F	Breast	WBRT SRS	37.5 24	L cerebellar	14 8		10q2w	7		25	77.3				8	0	8	Y	Improved	fatigue grade 2
	57	F	Fallopian tube	SRS	20	L parietal	6		15q3w	9		74.5	84.9				8	0	8	Y	Resolved	
	63	M	Rectal	WBRT SRS	37.5 16	L frontal	12 4		10q2w	8		25.4	13.5				4	2	2	Y	Resolved	
	67	F	NSCLC	SRS	18	L frontal	3		10q2w	8		22	53				4	0	4	Y	Resolved	
	45	M	NSTC	SRS	18	R frontal	5		5q2w	4		32.2	46.2				4	0	4	Y	Resolved	Hypertension grade 2
Xiang-Pan, L., et al. (2015) [49]	60	F	lung	WBRT SRS		L temporal	12		7.5q3w			⇓	⇓								Resolved	
Alessandretti, et al. (2013) [48]	51	F	melanoma	WBRT SRS (3 lesions)			17		5q2w/ 7.5q4w		3	⇓	⇓				4	0	4	severe drowsiness, unable to self-ambulate	Resolved	
	48	F	melanoma	SRS WBRT			6		5q6w			⇓	⇓				4	0	4	partial seizures (facial tremor)	Resolved	
Tanigawa, et al. (2019) [56]	78	M	Lung (adenocarcinoma)	STI (stereotactic irradiation)			9.2		15q3–4w			⇓	⇓							Y	Resolved	Hypertension, proteinuria
	74	M	Lung (adenocarcinoma)	STI (stereotactic irradiation)			12.2		15q3–4w			⇓	⇓							Y	Resolved	Hypertension
	49	F	Lung (adenocarcinoma)	STI (stereotactic irradiation)			5		15q3–4w			⇓	⇓							Y	Resolved	Oedema, hypertension, proteinuria
	44	F	Lung (adenocarcinoma)	STI (stereotactic irradiation)			4.6		15q3–4w			⇓	⇓							Y	Resolved	proteinuria
Ma, Y., et al. (2017) [57]	58	F	NSCLC	SRS			11		5 mg/kg q2w		4 weeks	⇓	⇓							speech disorder and weakness in the right arm	Improved	
	66	F	NSCLC	SRS			2		7.5 mg/kg q3w			⇓	⇓							headache and fatigue	resolved	
Glitza, I. et al. (2017) [58]	56	M	Melanoma	WB SRS	30 18	L frontal	4		7.5	4		⇓	⇓							Memory loss, seizure	Improvement	
	71	F	Melanoma	SRS	20	R frontal	13		7.5	3		⇓	⇓							Seizures, expressive aphrasia	Improvement	
	64	F	Melanoma	WB	30	R parietal	4		7.5	5		⇓	⇓							Weakness, gait disturbance, aphasia, memory loss	Resolution	Arthralgia, dysgeusia
	52	M	Melanoma	SRS WB	20/12/18 30	R frontal	13		5	2										Weakness, gait disturbance, cognitive deficit	worsened	
	65	M	Melanoma	SRS	20/16	L temporal	8		7.5	2										None	worsened	
	37	M	Melanoma	WB	30	Bifrontal	8		10	6		⇓	⇓							Behavioral changes, memory loss	improvement	
	55	M	Melanoma	SRS	24/21	R occipital/R frontal	7		7.5	4		⇓	⇓							Seizure, memory loss	Improvement	
	58 +/−10.6	M 22, F 32					11.7	15.5		5.7	3.29	57.4%	56.2%	56	75	23.75	10.4	1.6	9.08			

*Abbreviations*: *CT* Clinical trial, *No* No of patients, *M* Male, *F* Female, *WBRT* Whole brain radiotherapy, *SRS* Stereotactic radiosurgery, *SRT* Stereotactic radiotherapy, *EBRT* External beam radiotherapy, *RT* Radiotherapy, *FSRT* Fractionated stereotactic radiotherapy, *RN* Radiation necrosis, *BV* Bevacizumab, *Tx* Treatment, *NSCLC* Non-small cell lung cancer, *FT* Fallopian tube, *NSTC* Non-seminomatous testicular cancer, *MRI* Magnetic resonance imaging, *PET* Positron, emission topography, *q2w* Every 2 weeks, *Y* Yes, *R* Right, *L* Left

### Radiographic response

**Fig. 2:**
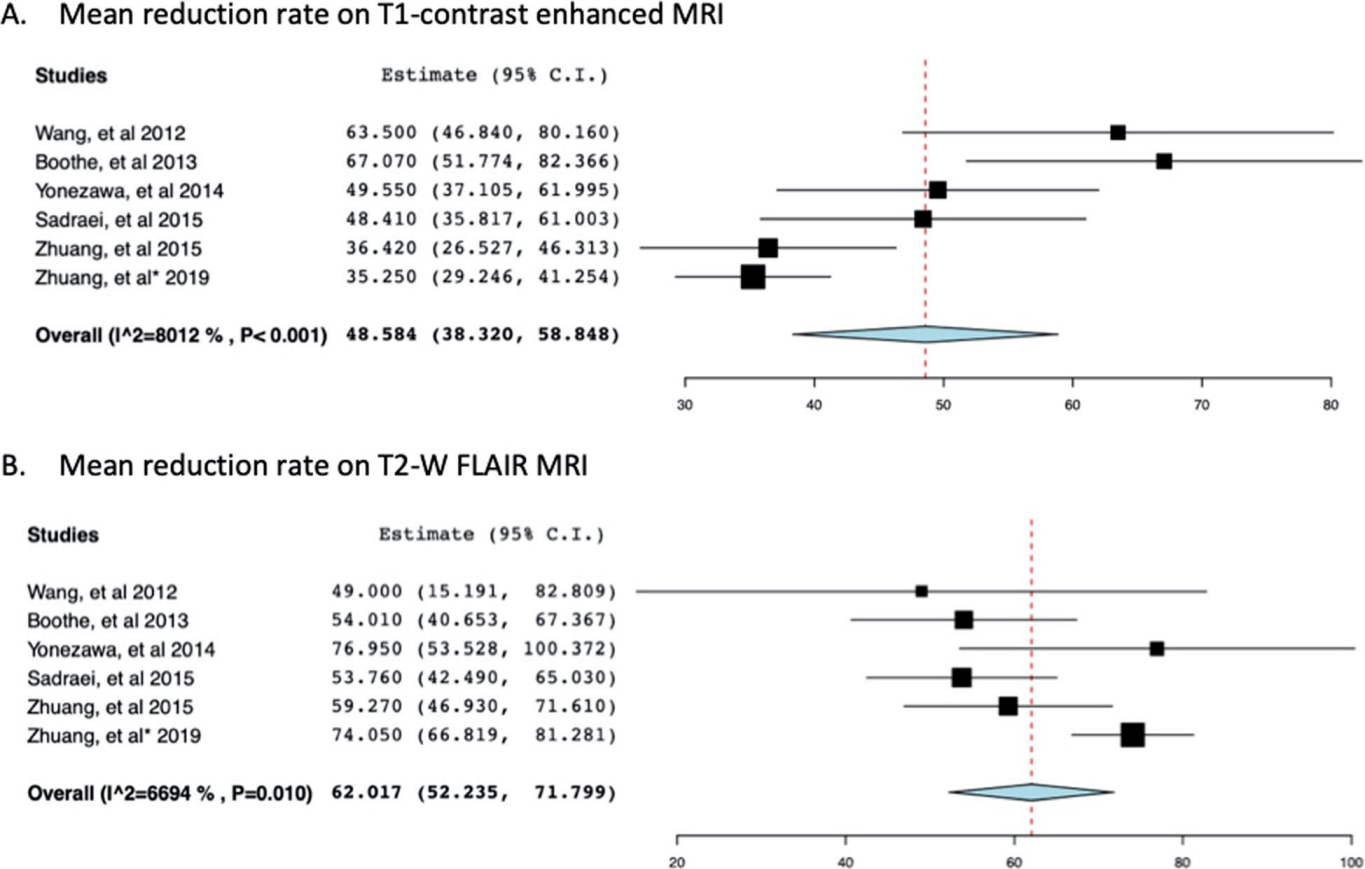
Forest plot of meta-analysis of mean reduction rate on T1-contrast enhanced MRI (**a**) and T2W FLAIR MRI (**b**) after bevacizumab (BV) treatment for radiation necrosis (RN) in patients with brain metastases

**Fig. 3:**
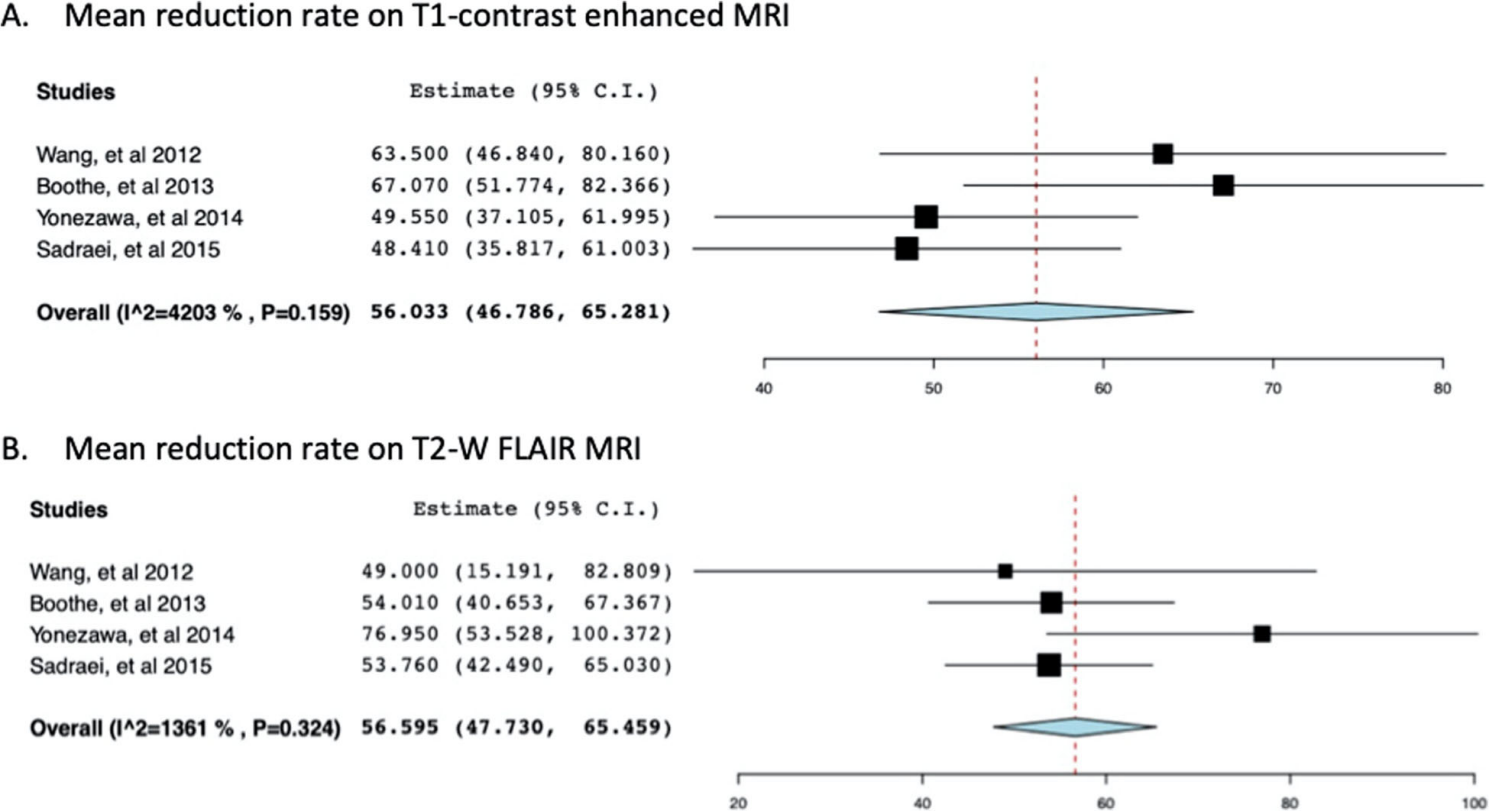
Sensitivity analysis of studies that used comparatively similar methods for estimation of reduction rates. Results are shown as forest plot of meta-analysis of mean reduction rate on T1-contrast enhanced MRI (**a**) and T2W FLAIR MRI (**b**)

Radiographic response was defined as any reduction observed in the RN or edema volume on MRI images (Gd-enhanced T1 and T2-FLAIR) [33, 48–58]. Radiographic response was 93% (*n* = 83) after BV therapy induction. Six (6.7%) patients experienced progression of RN or failed to respond to bevacizumab [33, 48–58]. Seven studies involving 73 patients with RN reported a mean volume reduction on T1-enhanced and T2-FLAIR MRI images (Table 4) [33, 50–55]. The weighted mean reduction in volume on T1 Gd-enhanced MRI was 47.03% (+/− 24.4), and on FLAIR imaging was 61.9% (+/− 23.3). The average decrease in volume reduction for each study is given in Table 4. The mean volume reduction for studies ranged between 35 and 63.5% on enhanced MRI and 49 and 75.1% on FLAIR MRI images [33, 50–55]. Pooling together the T1 and T2 MRI reduction rates by random effects model revealed a mean of 48.58 (95% CI: 38.32–58.85) for the T1 reduction rate and 62.017 (95% CI: 52.235–71.799) for T2W imaging studies (Fig. 2). Significant heterogeneity was revealed for both comparisons (*I*^*2*^ = 80%, *p* < 0.001; *I*^*2*^ = 66.9%, *p* = 0.01, respectively). We undertook sensitivity analysis by excluding the studies reported by Zhuang et al. as the method for data calculation differed from other studies [33, 55]. Heterogeneity was lost upon excluding the studies suggesting that the difference in calculation method may have been the contributing factor (Fig. 3). Analysis of individual patient data revealed a 57.4% mean volume reduction on T1 enhanced and 56.2% on flair imaging, for 41 patients (Table 3) [48–54, 56–58]. The extent of volume reduction on MRI images has not been reported in some studies [48, 49, 56–58].

**Table 4:** Radiographic responses and MRI changes after treatment with bevacizumab

Studies	No of patients	Radiographic responses	T1 Gd enhancement volume reduction (mean)	T2 FLAIR volume reduction (mean)
**Wang, et al. (2012)** [50]	5	4 (80%)	63.5%	49%
**Furuse, et al. (2013)** [52]	3	100%		75.1%
**Boothe, et al. (2013)** [51]	11	100%	67.1%	54.1%
**Alessandretti, et al. (2013)** [48]	2	100%		
**Yonezawa, et al. (2014)** [53]	2	100%	49.5%	76.9%
**Xiang-Pan, et al. (2015)** [49]	1	100%		
**Sadraei, et al. (2015)** [54]	17	16 (95.8%)	52%	53.7%
**Zhuang, et al. (2015)** [55]	14	13 (92.9%)	36%	59%
**Ma, Y., et al. (2017)** [57]	2	100%		
**Glitza, I. et al. (2017)** [58]	7	5 (71%)		
**Zhuang, et al. (2019)** [33]	21	20 (95.2%)	35%	74%
**Tanigawa, et al. (2019)** [56]	4	100%		
**This study**	89	83 (93%)	Mean: 47.03% (+/− 24.4)	Mean: 61.78% (+/− 23.2)

### Clinical improvement

Clinical improvement was measured in terms of improvement reported in neurological symptoms, KPS, and/or weaning of dexamethasone dosage [33, 48–58]. Overall, 85 patients presented with neurological symptoms because of RN, such as headaches, limb weaknesses, cognitive functions, and gait problems (Table 3) [33, 48–58]. After BV treatment, nine (10%) patients had stable symptoms, 39 (46%) patients had improved, and 34 (40%) patients had complete resolution of their symptoms [33, 48–56]. The symptoms worsened in three patients [50, 58]. Individual patient data was available for 54 patients [48–54, 56]. The KPS score was reported in 10 patients from three studies [50, 52, 53]. Improvement in KPS was observed in eight (80%) patients [50, 52, 53]. Dexamethasone discontinuation or reduction in dosage was observed in 30 (97%) of 31 patients who had recorded dosage before and after BV treatment [48, 50, 51, 54, 56]. The mean dose reduction for these patients was 9.08 mg (Table 3).

### Recurrence

Only one study (*n* = 14) reported a recurrence rate [55]. The recurrence rate was very high: 10 of the 13 responding patients had RN recurrence. Sadraei et al. also reported that four patients had RN recurrence, but the type of intracranial disease (primary brain tumor, NPC, or BM) was not identified [54]. A single patient in the study by Wang et al. also had recurrence with no evidence of intracranial disease type [50].

### Adverse events

Overall, five studies (*n* = 63) reported adverse events occurring in 14 (22%) patients after bevacizumab treatment (Table 5) [33, 54–56, 58]. A retrospective study reported grade 1 side effects in two (14%) patients. Adverse events reported were mild allergy and hypertension [55]. Hypertension resolved spontaneously. Similar side effects (mild allergy, hypertension) in two (9.5%) patients were reported in a prospective clinical trial conducted by the same group [33]. Side effects reported for individual patients were available in the study by Sadraei et al. [54]. One patient with non-small cell lung carcinoma (NSCLC) reported grade 1 proteinuria, for which bevacizumab treatment was withheld. Similarly, the other NSCLC patient reported a grade 2 urinary tract infection that also required withholding one dose of BV treatment. Of the 17 patients with RN, five (29%) patients (two with NSCLC, one with melanoma, one with breast cancer, and one with NSTC) reported side effects after BV treatment. Grade 3 deep venous thrombosis (DVT) and pulmonary embolism (PE) were observed in melanoma patients. The patient with breast cancer reported grade 2 fatigue, and the NTSC patient experienced grade 2 hypertension. All the participants in the case series (*n* = 4) reported by Tanigawa et al. experienced side effects involving hypertension, edema, and proteinuria [56]. Only one patient had experienced side effects such arthralgia and dysgeusia in the study by Glitza, I. et al. [58]. Adverse events were not reported in the remaining studies [48–53, 57].

**Table 5:** Adverse events reported with bevacizumab treatment

Studies	Patients	Symptoms
Sadraei, et al. (2015) [54]	5 (29%)	Grade 1: proteinuria (1). Grade 2: hypertension (1), fatigue (1), urinary tract infection (1). Grade 3: DVT/pulmonary embolism (1)
Zhuang, et al. (2015) [55]	2 (14%)	Grade 1: mild allergy (1), hypertension (1)
Zhuang, et al. (2019) [33]	2 (9.5%)	Grade 1: mild allergy (1), hypertension (1)
Tanigawa, et al. (2019) [56]	4(100%)	Hypertension (3), proteinuria (3), edema (1)
Glitza, I. et al. (2017) [58]	1(14%)	Arthralgia (1), dysgeusia (1)

## Discussion

We retrieved studies evaluating the efficacy of BV in the management of RN in patients who had received radiation therapy for brain metastases [33, 48–58]. Most patients showed a reduction in the edema and RN volume by over 50% on MRI images until their last follow-up [33, 48–56]. In some studies, edema volume reduction was over 70% in patients with BM [52, 53]. Radiographic responses corresponded with improvements in clinical outlook. Neurological symptoms were stabilized, improved, or completely resolved upon BV induction (Table 3). Several studies have reported a similar efficacy data for BV in patients with primary brain tumors (gliomas and glioblastoma), and NPC [31, 32, 45–47]. In a study by Wang et al., there were patients with other primary brain tumors who demonstrated a similar efficacy in reducing edema volume (T1 post-gd: 61%, T2 FLAIR: 57%), and showed improvement in neurological symptoms (100%) [50]. Fursue, et al. study, as well, had eight patients who had RN with primary brain tumors, other than the three BM patients [52]. A mean edema volume reduction rate of 45% was revealed for these patients. In addition to BM patients, seven other patients (five primary brain tumors and two arteriovenous malformations (AVM) patients) were also included in the study by Sadraei et al. [54]. The study reported an average reduction of 47.4 and 50.7% on both MRI images (T1W and FLAIR), respectively. Gonzalez et al. conducted a retrospective study showing radiographic and neurological symptom improvement in eight patients who had RN with primary brain tumors after being treated with BV (dosage: 5 mg/kgq 2 w /7.5 mg/kgq 3 w) [45]. Average reduction changes of 48 and 60% on post-contrast T1 and FLAIR MRI images were exhibited after a mean of 8.1 weeks from BV treatment start, respectively. In a separate retrospective study by Torcuator et al., six patients with RN diagnosed using biopsy and treated with BV also demonstrated significant reductions in both MRI images (T1 post-gd: 79%, T2 FLAIR: 49%) [46]. Li, et al., in their study comprising 50 NPC patients, though with a slightly lower response rate of 76.0% (38/50), had reported a significant decrease in edema volume reduction on FLAIR images (72.6%, *p* < 0.001) [47].

All these studies, however, constitute a low-level clinical evidence for the efficacy of BV therapy [45–47, 50, 52, 54, 56–58]. Zhuang et al. conducted a prospective clinical trial involving 21 patients who had RN with brain metastatic disease [33]. All patients, except for one, showed radiographic improvement. There is class I evidence for patients with primary brain and NPC tumors [31, 32]. Levin et al., in a randomized placebo-controlled trial, using a bevacizumab dose of 7.5 mg/kg every 3 weeks for seven patients with biopsy-proven RN with primary brain tumors, showed an average percentage change of 59 and 63% in RN volume on T1W and FLAIR images, respectively [31]. A recently concluded RCT involving 58 NPC patients treated with bevacizumab revealed a 65.5% (38/58) response rate [32]. The mean percentage change in RN volume observed on T1 post-gd and T2W FLAIR MRI were 25.5 and 51.8%, respectively. The mean change between before and after bevacizumab treatment was significant for both detected MRI images. Both these studies have reported significant differences in the radiographic responses and RN volume reduction rates observed on both MRI images between bevacizumab and placebo/corticosteroids, suggesting a better outcome for bevacizumab [31, 32].

In our systematic review, one study reported a very high RN recurrence rate (77%) in BM patients [55]. Other studies have failed to report recurrence of such a magnitude. Other than the two studies mentioned in the Results section, there are few other studies that also have cases of RN recurrence [50, 54]. Two patients in the RCT conducted by Levin et al. reported RN recurrence in glioma patients [31]. NPC patients from two other studies have also shown a moderate rate of recurrence [32, 47]. A recurrence rate of 39.5% was observed in a retrospective study of 50 NPC patients [47]. A similar recurrence rate (36.8%) was also demonstrated in the RCT of 58 NPC patients conducted by Xu et al. [32]. The underlying mechanism has not been exclusively investigated in these patients. Apparently, all three kinds of intracranial diseases (primary brain tumors, metastatic, or NPC) have registered RN recurrence [31, 32, 47, 55]. Recurrence was slightly higher in BM patients as reported, but the study had a low level of evidence. Hence, no conclusions could be drawn about the relationship between RN recurrence and the underlying intracranial disease type. Zhuang et al. identified a correlation between RN recurrence and duration after the initial BV withdrawal [55]. Further, Li et al. indicated that duration from induction of radiation therapy RN diagnosis and BV intervention as predictive factors for RN recurrence [47]. Further investigations are required to establish any underlying cause of RN recurrence. Another important aspect of RN recurrence is its diagnosis. Pathology is the standard for diagnosing RN or recurrence [76–79]. However, almost all of these studies relied on imaging criteria reported in previous studies for the diagnosis of RN and recurrence [31, 32, 47, 55, 76–79]. For example, in a case report, re-enlargement of RBN after being on BV for 8 months was attributed to recurrence of lung cancer as resected specimen revealed necrotic areas with viable tumor cells [80]. Hence, an accurate recurrence rate could only be determined with pathology, which could be further examined by larger comprehensively organized trials.

In this systematic review, clinical improvement was observed in a majority of the patients; however, some patients did not show any clinical improvement or experienced symptomatic worsening and progression. Medical literature also reveals similar examples. In the study by Gronier et al., no clinical improvement was observed in all three participants with malignant brain tumors after BV therapy (10 mg/kg per month) [81]. One patient had experienced lymphopenia after one perfusion of bevacizumab; the other had developed a transient ischemic attack and a corneal ulcer. Side effects reported in our review were mild, and only one grade 3 pulmonary embolism was described [33, 54–58]. Several other investigations have also highlighted similar low-grade adverse events [31, 32, 46, 50, 53]. In the retrospective study of Torcuator, et al. (*n* = 6), only one patient experienced mild fatigue after BV treatment [46]. Grade 2 AEs, including hypertension, fatigue, and proteinuria, were observed in 18% (3/17) of participants of the study by Wang, et al. [50]. However, the patients’ primary intracranial diseases were not identified. In the study by Yonezawa, et al., 33% (3/11) of participants had also shown grade 1 or 2 side effects such anemia, leukopenia, neutropenia, and lymphocytopenia [53]. More importantly, the class I evidence in this regard has shown the safety of BV therapy in primary brain tumors and NPC patients [31, 32]. Levin et al. reported that six (55%) patients experienced side effects [31]. Three of these adverse events were considered serious, including aspiration pneumonia, pulmonary embolus secondary to DVT, and superior sagittal sinus thrombosis. The other three patients showed ischemic changes due to small vessel thrombosis [31]. Another RCT conducted by Xu et al. reported 40 grade 1 or 2 adverse events experienced by 58 patients with NPC [32]. Only one grade 3 adverse event of ischemic stroke was observed. Furthermore, a similar portfolio was revealed for the corticosteroid-treated group, suggesting that BV treatment may not increase the toxicity experienced by patients with RN [32].

From the literature, it appears that bevacizumab was able to elicit therapeutic efficacy at any prescribed dose or frequency [31–33, 40–56]. The initial doses used were 5, 7.5, 10, and 15 mg/kg every 2 weeks to every 6 weeks. All doses were tolerated and were not associated with any increase in toxicity. It has been suggested that BV efficacy is associated with its anti-angiogenic effects rather than the dose [33]. In a case report, BV at a dose as low as 3 mg/kg was shown to be effective [48]. In a prospective clinical trial, patients were exposed to ultra-low doses of BV at 1 mg/kg [33]. Radiographic responses were observed in 20 of the 21 patients. Such a versatile dosing profile makes this treatment reachable to a broader population, as it is an expensive treatment. To date, exact cost-benefit relationship evaluation has not been adequately addressed for bevacizumab therapy [29]. It may cost around 4800 to 19,200 U.S. dollars (USD) for a single four to eight-week course of 5 to 10 mg/kg, administered every other week at a cost of 600 USD per 100 mg [82, 83]. An increase of 2.4 months in survival, a 20% improvement in a patient’s quality of life, or a linear combination of the two was required for bevacizumab treatment to be considered cost-effective according to a basic hypothetical calculation using 10,000 USD cost for a course of BV therapy and a quality-adjusted-life-year (QALY) threshold of 50,000 USD [84]. Hence, further studies are needed to establish a dose requirement for achieving the maximum benefit and to make the bevacizumab treatment cost-effective.

Several observations limit the results of our study. As a systematic review, the incorporated data comes from heterogeneous populations, diverse treatment centers, and a variety of research designs used for investigations. Moreover, the time period in which the case reports/studies were undertaken also varied. We included case reports and some retrospective studies [48–56]. Retrospective studies are prone to selection bias, recall bias, or misclassification bias and are subject to confounding [85]. Most of these studies mainly constitute class III level evidence, except for two prospective studies [48–56]. The types of radiation also differed from patient to patient. Moreover, pathology reports are used as standard for the diagnosis of RN; however, these studies mostly used imaging studies for RN diagnosis [48–56]. Some of the studies reported global adverse events/recurrence rates without differentiating between tumor types; however, they also contained participants other than BM patients [50, 53, 54]. Nonetheless, we presented the recurrence rates in results and side effects in the Discussion section to construct a better recurrence rate/adverse event profile for the readers. The follow-up for different studies also varied. The likelihood of only BV-responding patients being included in the study may also be prone to publication bias.

## Conclusions

According to our results, bevacizumab can be considered safe and efficacious for BM patients diagnosed with RN. However, the level of evidence presented was low, making our bevacizumab efficacy results inconclusive. Furthermore, several dimensions of BV treatment for RN were less clarified and should be investigated in future trials. These include the diagnosis standard used for RN, impact of type/dose/fractionation of radiation therapy used on RN, patterns, and underlying mechanism of recurrence. The pending results of a phase II trial (NCT02490878) of BV plus corticosteroids versus corticosteroids plus placebo for radiation necrosis after radiosurgery for brain metastases will further define the role of bevacizumab in the management of radiation necrosis.

## Data Availability

The datasets used and/or analysed during the current study available from the corresponding author on reasonable request.
